# Sarcoid reaction in the spleen after sigmoid colon cancer resection: a case report

**DOI:** 10.1186/s40792-016-0244-4

**Published:** 2016-10-18

**Authors:** Takafumi Shima, Yoshinori Tanaka, Kunihiro Katsuragi, Nagahisa Fujio, Shuichi Nakatani, Yasutsugu Kobayashi, Tadayuki Hida

**Affiliations:** 1 Department of Surgery, Minami Osaka Hospital, Osaka, Japan; 2 Department of Pathology, Minami Osaka Hospital, Osaka, Japan

**Keywords:** Sarcoid reaction, Colon cancer, Spleen

## Abstract

**Background:**

A sarcoid reaction is a phenomenon characterized by histologically proven granulomatous lesions without evidence of sarcoidosis. This pathology is a benign tumor itself, but several reports have described sarcoid reactions accompanying malignant tumors. Sarcoid reactions occur in various cancers, such as skin, lung, ovary, stomach, and breast cancers. However, only a few published reports have described sarcoid reactions in patients with colorectal cancer.

**Case presentation:**

A 76-year-old woman underwent laparoscopic sigmoidectomy for sigmoid colon cancer. The postoperative follow-up computed tomography and 18-fluorodeoxyglucose positron emission tomography–computed tomography findings were suspicious for splenic metastasis of the sigmoid colon cancer. The patient then underwent laparoscopic splenectomy. Histopathological examination of the resected lymph nodes and spleen showed a non-caseating epithelioid cell granuloma. The patient was diagnosed with a sarcoid reaction.

**Conclusions:**

To our knowledge, this is the first report of a sarcoid reaction in the spleen and regional lymph nodes after colon cancer resection. The effect of a sarcoid reaction on the prognosis in patients with colorectal cancer has not been fully determined because of the small number of such cases. Further analyses involving a larger number of cases are necessary to evaluate the relationship between sarcoid reactions and prognosis in patients with colorectal cancer. We herein present an extremely rare case of a sarcoid reaction in the spleen and regional lymph nodes.

## Background

A sarcoid reaction is a phenomenon characterized by histologically proven granulomatous lesions without evidence of sarcoidosis. Many malignancies have been associated with sarcoid reactions [1]. However, a sarcoid reaction in a patient with colorectal cancer is rare. We herein report an extremely rare case of a sarcoid reaction in the spleen and regional lymph nodes that presented 19 months after sigmoidectomy.

## Case presentation

**Fig. 1:**
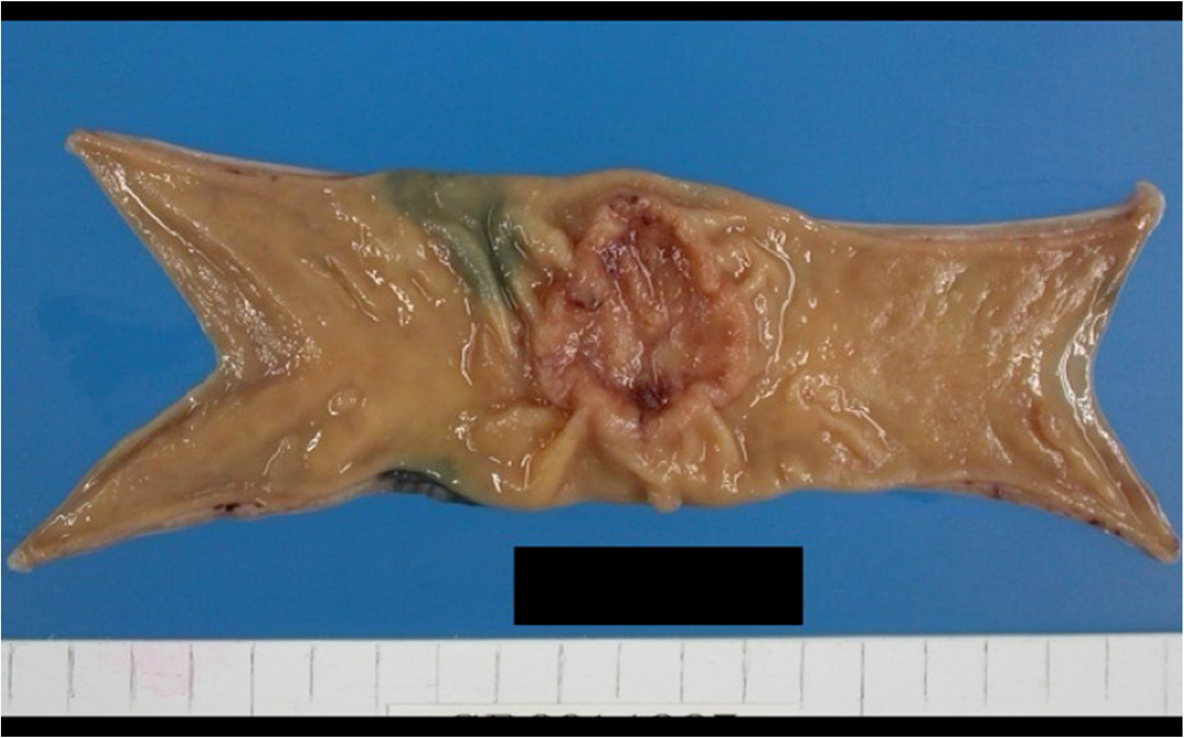
Operative specimen findings. Operative specimen revealed a sigmoid colon tumor measuring 35 × 40 mm

**Fig. 2:**
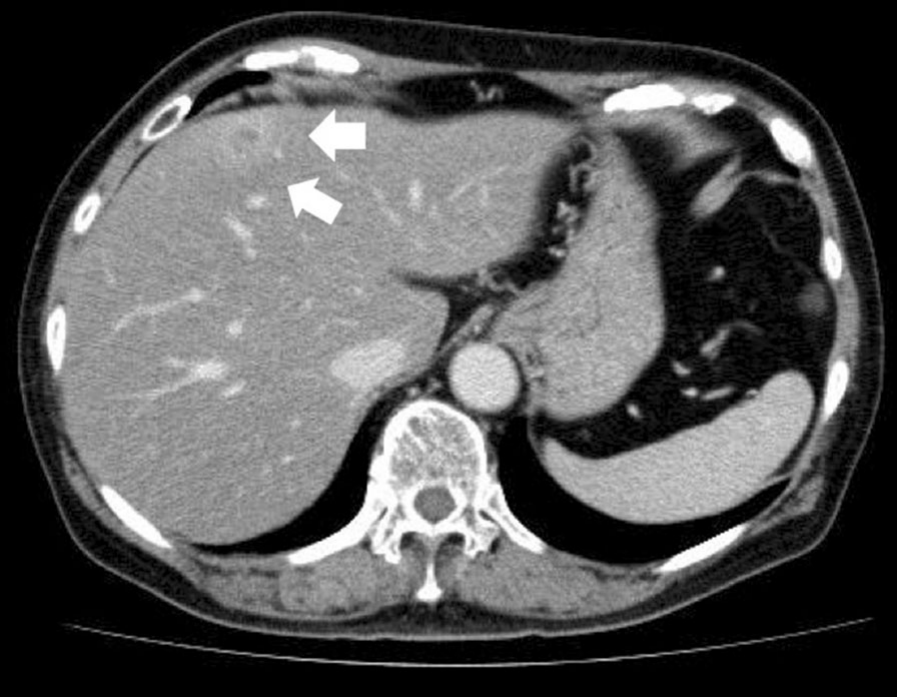
CT scan findings. CT scan revealed a 10-mm space-occupying lesion in the liver with ringed enhancement (*arrows*)

**Fig. 3:**
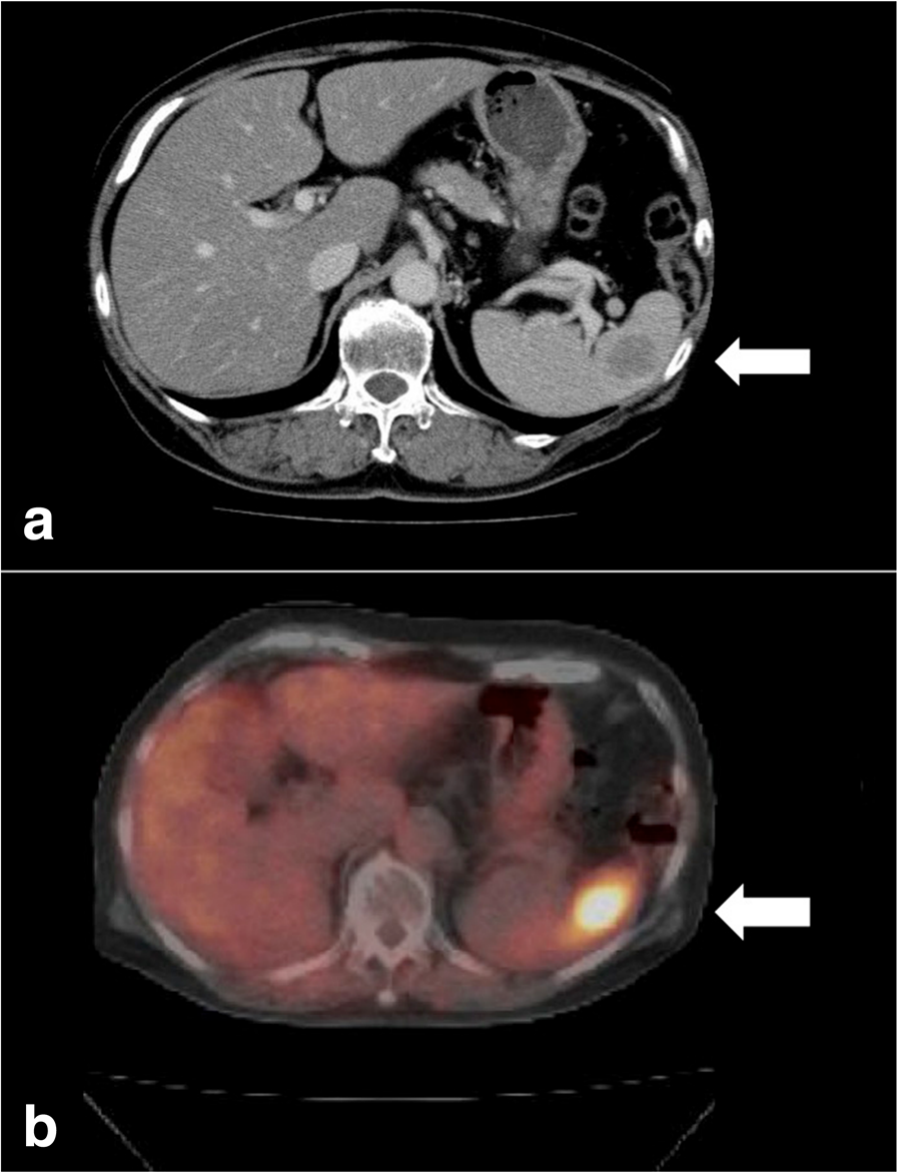
CT scan and FDG PET-CT findings. **a** CT scan revealed a 25-mm low-density area in the spleen (*arrow*). **b** FDG PET-CT revealed hypermetabolic lesions in the spleen (*arrow*)

The patient was a 76-year-old woman. She underwent laparoscopic sigmoidectomy for sigmoid colon cancer in February 2014. An operative specimen revealed a sigmoid colon tumor measuring 35 × 40 mm (Fig. 1). Histopathological examination of the tumor showed tubular adenocarcinoma (pT3, pN2 (#214(5/12), #242(0/9), #252(0/2), #253(0/2)), M0, H0, P0, PUL0, pStgaeIIIb, D3, pPM0, pDM0, RM0, R0, CurA, tubular adenocarcinoma, well-differentiated type, intermediate, INFb, ly0, v0, PN0). She was then started on adjuvant chemotherapy comprising a combination of tegafur, gimeracil, and oteracil potassium (TS-1). Six months after starting TS-1, computed tomography (CT) revealed a liver metastasis (Fig. 2). After seven cycles of folinic acid, 5-fluorouracil, and oxaliplatin (FOLFOX) with bevacizumab, the patient underwent laparoscopic partial hepatectomy. She received adjuvant chemotherapy (capecitabine) for 6 months postoperatively. In September 2015, CT revealed a 25-mm low-density area in the spleen (Fig. 3a). Additionally, 18-fluorodeoxyglucose positron emission tomography (FDG PET)-CT revealed hypermetabolic lesions in the spleen (maximum standardized uptake value of 3.4) (Fig. 3b). Laboratory data showed no abnormalities. There was no elevation of the tumor markers carcinoembryonic antigen or cancer antigen 19-9. Chest X-ray showed no evidence of lymphadenopathy. We therefore suspected that the splenic mass was a metastasis from colon cancer. For the cure of isolated splenic metastasis, we performed laparoscopic splenectomy in March 2015.

**Fig. 4:**
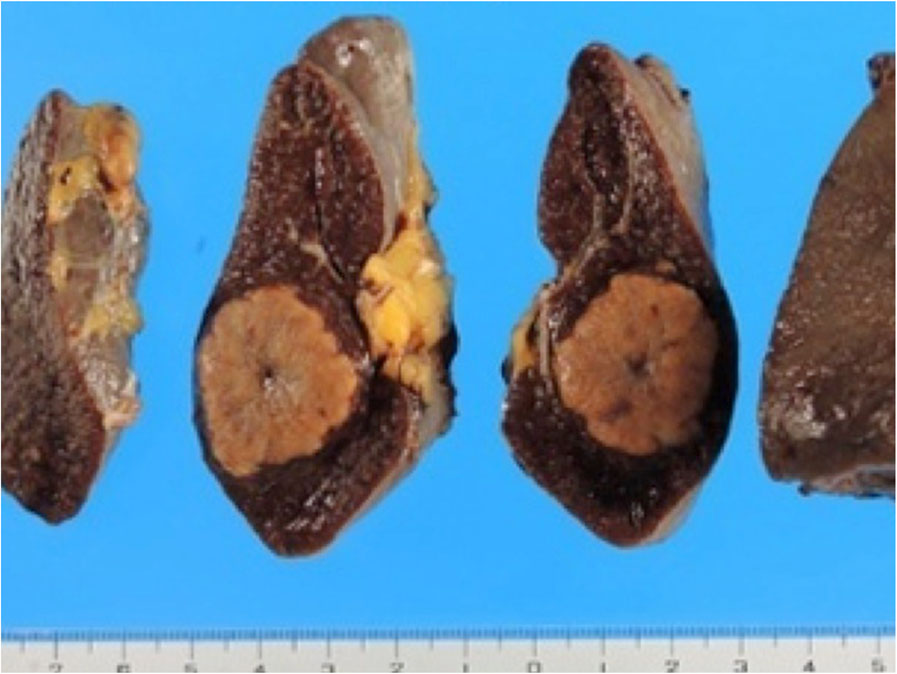
Operative specimen findings. Operative specimen revealed a splenic tumor measuring 28 × 22 mm

**Fig. 5:**
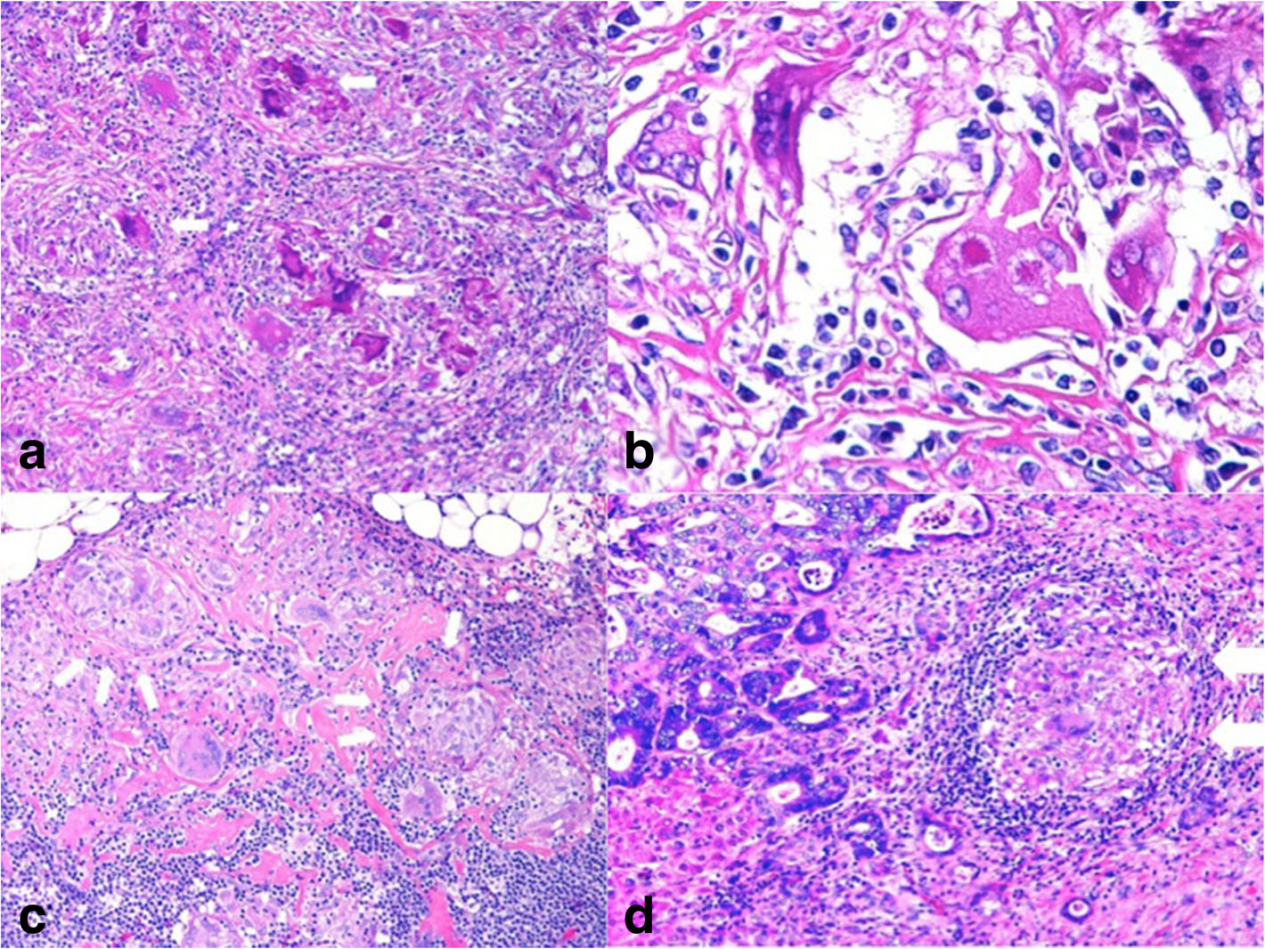
Histopathological findings. **a** Histopathological findings in the dissected spleen and lymph nodes showed epithelioid cell granulomas with polynuclear giant cells, but no central caseous necrosis (*arrows*). **b** Asteroid bodies were present (*arrows*). **c** The findings in the lymph nodes were similar to those in the spleen (*arrows*). No evidence of metastatic carcinoma was found. **d** The retrospective histopathological findings in the part of dissected liver metastasis showed epithelioid cell granulomas with polynuclear giant cells

An operative specimen revealed a splenic tumor measuring 28 × 22 mm (Fig. 4). Histopathological examination of the resected lymph nodes and spleen showed a non-caseating epithelioid cell granuloma (Fig. 5a–c) in which no tumor cells were identified. The retrospective histopathological examination of the primary sigmoid colon cancer with region lymph node and metachronous liver metastasis revealed sarcoid reaction in the part of dissected liver metastasis (Fig. 5d) such as the resected lymph nodes and spleen findings. Four months postoperatively, CT revealed a new liver metastasis. At the time of this writing, the patient was undergoing chemotherapy.

## Conclusions

Sarcoidosis is a systemic disease of unknown cause. It produces epithelioid granulomas in one or several body sites. In patients with other diseases, similar epithelioid granulomas are formed in the affected organ itself and in regional lymph nodes, and the formation of such granulomas is termed a sarcoid reaction [1]. An association between sarcoid reactions and malignancy has been reported several times [2]. Sarcoid reactions occur within the locoregional lymph nodes that drain the cancer. Brincker et al. [1] reported that sarcoid reactions occur in 4.4 % of patients with various cancers, such as skin, lung, ovary, stomach, and breast cancers. However, only a few published reports have described sarcoid reactions in patients with colorectal cancer [3–6]. Furthermore, there are no reports of this reaction in the spleen and regional lymph nodes after colon cancer resection. In the present case, we suspected splenic metastasis while following up our patient for colon cancer. The CT and FDG PET-CT findings were not inconsistent with splenic metastasis. However, it is extremely difficult to differentiate between malignancy and a sarcoid reaction using CT or FDG PET-CT because the tissue involved in the sarcoid reaction accumulates FDG. When a sarcoid reaction occurs in the mediastinal lymph nodes, endoscopic ultrasound with fine-needle aspiration of the lymph nodes is believed to be the best choice for diagnosis [5]. However, the performance of a splenic biopsy to confirm the diagnosis is unrealistic because of the risk of peritoneal dissemination or bleeding. Therefore, when a sarcoid reaction occurs in the spleen, it is almost impossible to distinguish it from metastasis unless a surgical operation is performed. We suspected that the splenic mass was isolated metastasis from colon cancer at first. Splenic metastasis from colorectal cancer is generally a part of systemic disease. Whether to perform splenectomy in patients with isolated splenic metastasis from colorectal cancer is controversial. However, Abi saad et al. [7] reported that splenectomy for isolated splenic metastasis can achieve long-term survival. Furthermore, Jiddou et al. [8] reported 31 cases of isolated splenic metastasis from colorectal cancer, and splenectomy was performed in all cases. In addition, there are reports of laparoscopic approach too [8, 9]. Therefore, we performed laparoscopic splenectomy for the cure of isolated splenic metastasis, and yet histopathological examination of the spleen showed a sarcoid reaction. A sarcoid reaction is a benign tumor itself. When splenic metastasis from colon cancer is suspected, the possibility of a sarcoid reaction should be considered. Minimally invasive surgery such as laparoscopic surgery may therefore be the best option for diagnosis and treatment, as in our case.

The pathogenesis of a tumor-associated sarcoid reaction in the lymph nodes or spleen has not yet been determined. Fujii et al. [4] reported that the possible mechanisms of such a reaction are summarized as follows: (1) a localized defense reaction to the tumor cells themselves, (2) a simple tissue reaction to a tumor embolism in the lymphatic system or capillaries, and (3) an immunological reaction to substances released from the tumors transported along the lymphatic system. Our patient developed a sarcoid reaction in the spleen and regional lymph nodes after adjuvant chemotherapy for colon cancer; moreover, this reaction was recognized in part of liver metastasis by retrospective study. The mechanism of a sarcoid reaction after primary sigmoid colon cancer resection remains a mystery. However, from that, a sarcoid reaction contains in part of liver metastasis; it is speculated that carcinoma cells remaining at the cellular level in the body received some effect by repeated chemotherapy and it showed sarcoid reaction. We concluded that the chemotherapy gave rise to an immunological reaction in our case.

Brincker [1] reported that a sarcoid reaction is most likely caused by antigenic factors against metastatic extension. Pavic et al. [10] suggested that a sarcoid reaction may be associated with a better prognosis in patients with gastric cancer. In another study, however, patients with gastric cancer who developed a sarcoid reaction in both the regional lymph nodes and spleen were more frequently in the advanced stages of disease than were patients with a sarcoid reaction in the regional lymph nodes alone [11]. Even in our case, the sarcoid reaction occurred in both the regional lymph nodes and spleen. Our patient exhibited a new liver metastasis on CT 4 months after the splenectomy. Therefore, we consider that the prognosis of colorectal cancer with a sarcoid reaction in the regional lymph nodes and spleen may be poor, as in patients with gastric cancer. However, to fully determine the relationship between a sarcoid reaction and the prognosis of patients with colorectal cancer, an investigation involving a larger number of cases is required.

In summary, we have reported a rare case of a sarcoid reaction in the spleen and regional lymph nodes. The effect of a sarcoid reaction on the prognosis in patients with colorectal cancer has not been fully determined because of the small number of such cases. Further analyses involving a larger number of cases are necessary to evaluate the relationship between sarcoid reactions and prognosis in patients with colorectal cancer.

## Abbreviations

CT: Computed tomography
FDG: 18-Fluorodeoxyglucose
FOLFOX: Folinic acid, 5-fluorouracil, and oxaliplatin
PET: Positron emission tomography
TS-1: Tegafur-gimeracil-oteracil potassium combination S-1
